# Surveillance of Domestic Violence Using Text Mining Outputs From Australian Police Records

**DOI:** 10.3389/fpsyt.2021.787792

**Published:** 2022-02-09

**Authors:** George Karystianis, Armita Adily, Peter W. Schofield, Handan Wand, Wilson Lukmanjaya, Iain Buchan, Goran Nenadic, Tony Butler

**Affiliations:** ^1^School of Population Health, University of New South Wales (NSW), Sydney, NSW, Australia; ^2^Hunter New England Local Health District, Newcastle, NSW, Australia; ^3^The Kirby Institute, University of New South Wales, Sydney, NSW, Australia; ^4^School of Computer Science, University of Technology, Sydney, NSW, Australia; ^5^Institute of Population Health, University of Liverpool, Liverpool, United Kingdom; ^6^School of Computer Science, University of Manchester, Manchester, United Kingdom

**Keywords:** domestic violence, text mining, surveillance, public health, mental illness

## Abstract

In Australia, domestic violence reports are mostly based on data from the police, courts, hospitals, and *ad hoc* surveys. However, gaps exist in reporting information such as victim injuries, mental health status and abuse types. The police record details of domestic violence events as structured information (e.g., gender, postcode, ethnicity), but also in text narratives describing other details such as injuries, substance use, and mental health status. However, the voluminous nature of the narratives has prevented their use for surveillance purposes. We used a validated text mining methodology on 492,393 police-attended domestic violence event narratives from 2005 to 2016 to extract mental health mentions on persons of interest (POIs) (individuals suspected/charged with a domestic violence offense) and victims, abuse types, and victim injuries. A significant increase was observed in events that recorded an injury type (28.3% in 2005 to 35.6% in 2016). The pattern of injury and abuse types differed between male and female victims with male victims more likely to be punched and to experience cuts and bleeding and female victims more likely to be grabbed and pushed and have bruises. The four most common mental illnesses (alcohol abuse, bipolar disorder, depression schizophrenia) were the same in male and female POIs. An increase from 5.0% in 2005 to 24.3% in 2016 was observed in the proportion of events with a reported mental illness with an increase between 2005 and 2016 in depression among female victims. These findings demonstrate that extracting information from police narratives can provide novel insights into domestic violence patterns including confounding factors (e.g., mental illness) and thus enable policy responses to address this significant public health problem.

## Introduction

The World Health Organization (WHO) defines public health surveillance as “an ongoing, systematic collection, analysis and interpretation of health-related data” that is essential to the planning, implementation, and evaluation of public health practice (1). Surveillance is undertaken to inform prevention and control measures and can serve as an early warning system, identify public health emergencies, document the impact of an intervention or progress toward the identification of public health targets and goals and contribute to a better understanding of the problem.

Domestic violence is recognized as a significant public health problem that mostly affects women. In Australia, the Personal Safety Survey reports that 39% of the population aged more than 15 years have experience physical or sexual violence perpetrated by a current or a former partner (2). On average one woman per week is murdered by a current or former intimate partner, and 1 in 6 women and 1 in 16 men have been subjected to physical and/or sexual violence by a current or former partner (3). Reporting on domestic violence in Australia is based mostly on data from hospital presentations, court outcomes (e.g., domestic violence convictions), periodic surveys such as Australian Bureau of Statistics' Personal Safety Survey, and police records (3).

While these sources of information are important and allow a factual picture of domestic violence in Australia to be presented from the relevant agency perspective (i.e., hospital system, courts, police activity), there are many aspects and questions that remain unenumerated which could be helpful in the development of a more comprehensive picture of domestic violence (2, 4). This finer-grained information is traditionally provided by qualitative research collected from one-on-one interviews with victims or focus groups to support quantitative data yet is often critiqued due to the small sample size, selection bias, and the time required to conduct such research. In addition, despite the integration of national resources to help build a picture of domestic violence in Australia, many of the available data sources focus on violence perpetrated by a male intimate partner against a female victim (4).

Data on the characteristics of victims and perpetrators is often lacking from domestic violence reporting such as minority group status (e.g., people with disabilities, veterans, immigrants, and the LGBTQ+ status) (4–6). Similarly, absent is the perceived [by the police] role of licit or illicit drugs, mental health status of victims and perpetrators, the cause of the event (e.g., accusations of infidelity), injuries (apart from those requiring hospital visits), reports of coercive controlling behaviors, stalking, abuse type, and threats of further violence toward a victim. Access to such information on domestic violence events would be extremely valuable in terms of providing more context specific information to inform policy development, service delivery, and the provision of more targeted services.

In New South Wales (NSW), the first point of contact for many individuals involved in domestic violence occurs when the police attend an event. During these interactions with victims and perpetrators, personal and demographic details are recorded as structured data (called “fixed fields”) and entered into the Computerized Operational Policing System (COPS) (e.g., name, age, sex, ethnicity), the type of the offense (e.g., assault, malicious damage), the relationship between perpetrator and victim, as well as spatio-temporal information (e.g., date, postcode, premises type). In addition, to the fixed fields, a free text narrative is also written by the attending police officer(s) describing the event covering information such as the mental health status of perpetrators and victims, recorded abuse types, and injuries, threats of future violence, weapons, role of licit and illicit substances, and the alleged cause(s) of the event.

While the text narratives can be used in subsequent court proceedings, the rich information they contain is rarely been used for surveillance and research purposes. This is possibly due to the strict access protocols involved in researchers accessing data, a lack of awareness of their potential, and the limited use of text mining by epidemiologists for surveillance and monitoring purposes. However, easier access to automated methods can now deliver sophisticated approaches for large scale processing of free text such as text mining, that can harvest salient information quickly and reliably. Text mining has been used for the past 30 years to identify concepts of interest from unstructured text in fields such as medicine to fill in gaps in missing information or to provide new insights that were not previously available (7, 8). Few attempts have utilized text mining on police data and these have either involved small samples (9–14) or focused on data classification (15).

We recently demonstrated the successful application of text mining to a large corpus of police domestic violence event narratives to identify mentions of mental illness, abuse type(s), and victim injuries (16, 17). We also demonstrated that the extracted information can be used to provide insights into domestic violence and mental illness (18) and in the context specific diagnoses (i.e., autism) (19), the setting (i.e., nursing homes) (20), and abuse type (i.e., non-fatal strangulation) (21).

In this paper we use our text mining pipeline to analyze a population-level corpus of police domestic violence narratives from January 2005 to December 2016 to demonstrate its use for surveillance of domestic violence in NSW.

## Materials and Methods

### Data

The New South Wales Police Force (NSWPF) made available 492,393 police recorded domestic violence event narratives from January 2005 to December 2016 that were flagged in the fixed fields with one of the following tags: “domestic” as the type of offense, “domestic violence related” as the associated factor of the police event; or the relationship status between the victim and the person of interest (POI—an individual suspected/charged with a domestic violence offense) being described as “spouse/partner (including ex-spouse/ex-partner),” “boy/girlfriend (including ex-boy/ex-girlfriend),” “parent/guardian (including step/foster),” “child (including step/foster),” “sibling,” “other member of family (including kin),” or “carer.” The dataset also contained cases where no crime was committed but the police did attend the event.

Permission to access the police recorded domestic violence events was granted by the NSWPF following ethics approval from the University of NSW Human Research Ethics Committee (HC16558).

### Text Mining Approach

A text mining method was designed and implemented with GATE (General Architecture for Text Engineering) (22), a suite of tools that can be used for various natural language processing tasks such as information extraction. The implemented approach included the engineering of rules based on common syntactical patterns observed in the narratives from a sample of 200 event narratives that mentioned a mental illness (e.g., “the perpetrator was diagnosed and suffered for 10 years from paranoid schizophrenia”), abuse type(s) (e.g., “the perpetrator attempted to punch and slap the victim in the face”), and reports of victim injuries (e.g., “after inspecting the victim, the victim had suffered cuts and bruises in her arms”) (16, 17). We focused on these three attributes to highlight mental health conditions, the wide range of abuse types, and injuries sustained by victims from this population-based sample.

The rules make use of specific semantic anchors for victims (e.g., victim, person in need of protection) and POIs (e.g., person of interest, POI, perpetrator) to assign the extracted mention of a mental illness to a victim or a POI respectively. For the identification of abuse types, we crafted rules that included lexical patterns that specifically refer to the perpetration of violence from the POI (e.g., “POI attempted to stab the victim”). Similarly, rules were engineered for lexical patterns that suggest a sustained victim injury as a direct result of a POI's abuse (e.g., “victim sustained severe cuts from the offender's actions”). Dictionaries were manually crafted containing terms, common synonyms and abbreviations for mental illnesses, abuse types and injuries (16, 17).

The rules were fully evaluated against a sample of 100 event narratives for mentions of mental illness, abuse types and victim injuries. The sample was manually annotated twice by two experts (one in psychiatry and one in domestic violence) for the identification of mental illness mentions; and by two additional experts (one in psychiatry and one with background in medicine) for the identification of abuse types and victim injuries. The annotation process was done before the creation of any rules. To ensure consistency between the domain experts' annotations, the inter-annotator agreement was calculated as the absolute agreement rate (23), which resulted in 90% and 91% score for the two annotation tasks respectively (16, 17).

The returned precision of the methodology was >85% (i.e., the percentage of correctly identified mentions against the total number of identified mentions, a denominator that includes both true positives and false positive mentions identified by the rule-based approach); 97.5% for the identified mental illness mentions for POIs; 87.1% for those related to victims; 90.2% for the abuse types; and 85.0% for the victim injuries. Recall (i.e., the percentage of correctly identified mentions against the true positive mentions and false negative mentions by the rule-based approach) was 79.0% for the mental health mentions of POIs and victims, 89.6% for abuse types and 86.3% for victim injuries respectively. This resulted in F1-scores (i.e., a harmonic mean between precision and recall) being >80.0% (81% and 87% for the mental illness mentions for victims and POIs respectively; 89.8% for abuse types and 85.6% for victim injuries). Further details of the methodology (including error analysis) have been published elsewhere (16, 17).

We classified the returned abuse types into 44 different categories ranging from physical forms (e.g., punching, kicking) to psychological (e.g., intimidation) and social (e.g., limited access to children, social restrictions) (Supplementary Table 1). Injuries were classified into a total of 17 common types [scratching, grazing, red mark(s), tearing off (nail), bruise(s), cut(s), swelling, lump, unspecified injury, fracture, periorbital hematoma (aka black eye), broken tooth, burn mark, stab wound, bite mark, soreness, and bleeding] (17). The extracted mental illness mentions ranged from general descriptions (e.g., mood disorder, behavioral problems) to very specific terms (e.g., oppositional defiance disorder, paranoid schizophrenia). To be able to conduct analysis of the identified mental health mentions, we mapped them to the World Health Organization's International Classification of Diseases (ICD-10) Mental and Behavioral Disorders categories using four levels (16, 24) (Supplementary Table 2). When cases had ambiguous mapping, an experienced behavioral neurologist (PWS) was used to map the description to an appropriate ICD-10 code. Level 1 mapping included 18 ICD-10 broad categories with eight additional customized ones; four categories where a mental illness was implied through a particular medication or drug class (e.g., Zoloft, antidepressants); and four categories that covered “drug prescription abuse,” “substance abuse (unspecified),” “traumatic brain injury,” and “unspecified drug induced disorders.”

Cases in which the victim or the POI had an unknown mental illness, or an unknown drug-induced mental disorder, were assigned into the categories of “unspecified mental disorder” and “unspecified drug induced disorder” respectively. Cases in which mental illness mentions were very specific were mapped to lower-level ICD-10 categories (e.g., postpartum depression was mapped at the third level according to the ICD-10 schema). Because the mention has a third-level mapping, this indicates that it can also be mapped to the second (major depressive disorder, single episode) and first ICD-10 level (mood disorders). For reporting purposes, we show only events with mental illness at the second level of ICD-10 since the first level ICD-10 categories are too broad while mappings to third level codes were infrequent such that conditions like post-traumatic stress disorder and paranoid schizophrenia would not appear in the results.

This dataset of events can have more than one POI or victim involved. However, the implemented text mining methodology was unable to associate the extracted “mention” to a specific POI or victim, if more than one individual POI or victim were present in the domestic violence event. Thus, in the current analysis, results are presented only for events that involved a single POI against a single victim. This resulted to a total of 416,441 events out of 492,393 (84.5%).

We conducted bivariate comparisons of certain characteristics (i.e., injury and abuse types) by gender of victims and POIs using Chi-square tests [i.e., 2 by 2, Pearson's chi-squared test, with degrees of freedom of 1 = (2–1) × (2–1)]. We also conducted tests for trend analysis to assess linear increasing (or decreasing) trends in certain characteristics (i.e., abuse types, injury types, recorded mental illnesses) during the 2005–2016 period through the Cochran-Armitage test for trend test (25). Year was the ordered categorial variable representing calendar years, and binary outcome variables were the presence or absence of the events under investigation. Each comparison had a degree of freedom of 11 = (2–1) × (12–1).

## Results

**Figure 1:**
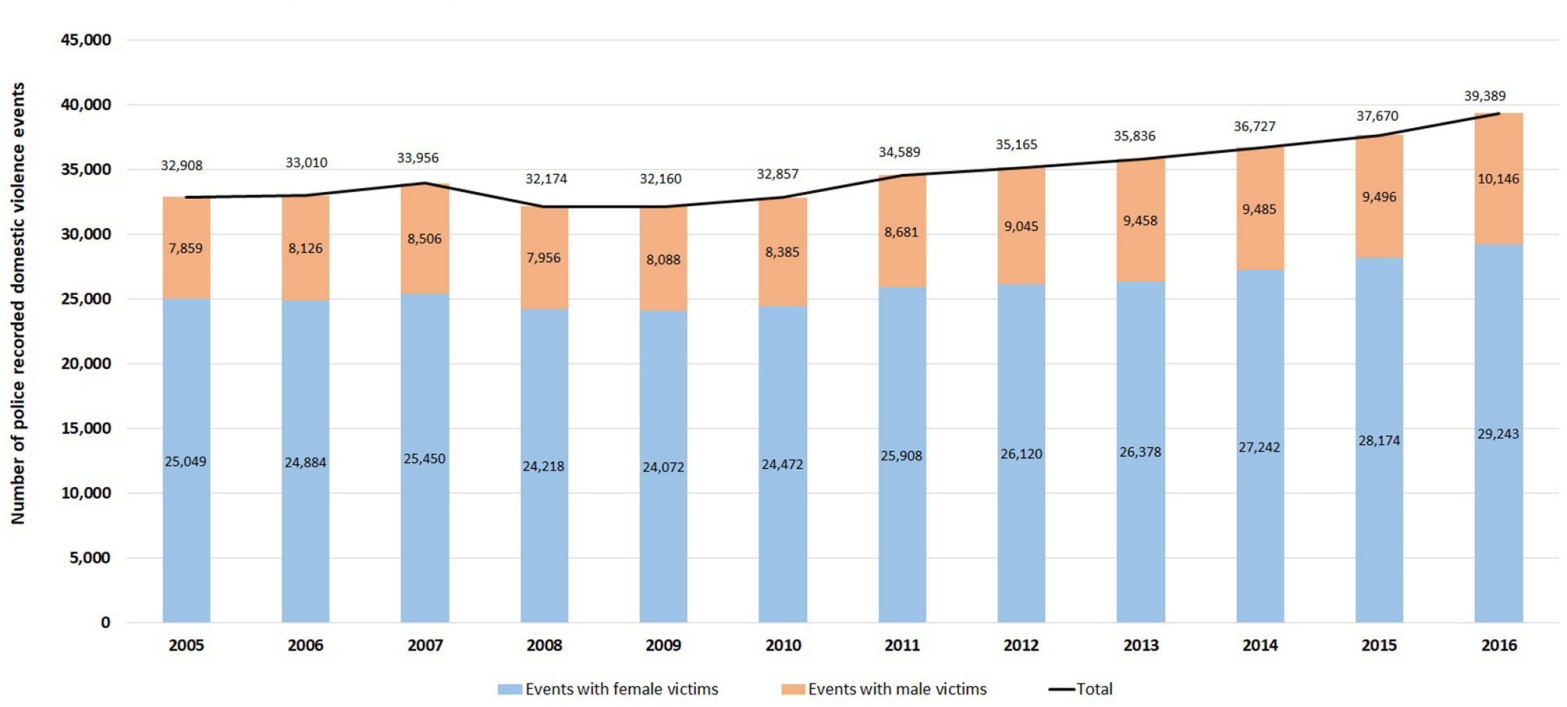
Yearly number of police recorded domestic violence events in NSW, January 2005–December 2016.

The NSWPF police attended 416,441 domestic violence events between 2005 and 2016 involving a single POI against a single victim. Of these, three-quarters of events (76.3%; 311,210) involved a female victim while 23.6% (96,228) involved a male victim; 2.1% of the events did not have a recorded gender for the victim. Almost 80% of the events involved a male POI (329,906) with 17.8% (74,323) having a female POI. From 2005 to 2016, while the distribution of male and female victims in domestic violence events increased over time, the increase in the proportion was larger for male victims (22.5% vs. 14.3% for female victims) (Figure 1). The total numbers of unique POIs and victims (i.e., one individual might be a victim or a POI in more than one event) were 214,185 and 244,219 respectively, with 22.8% (48,872) being female POIs unique to one event and 70.0% (195,347) being female victims unique to one event. One third (34.3%; 73,575) of POIs were involved in more than one domestic violence event.

### Setting

**Figure 2:**
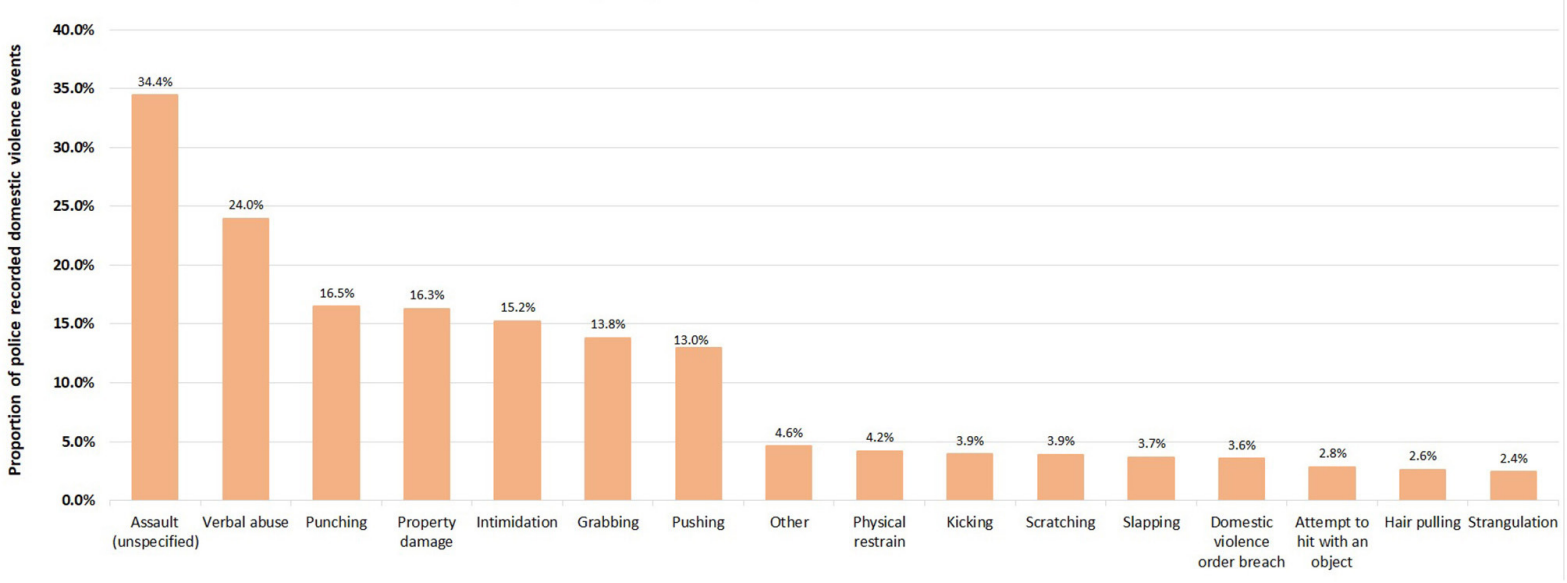
Proportion of abuse types in 353,161 police recorded domestic violence events that occurred in residential premises in NSW, January 2005–December 2016.

Almost four out of five domestic violence events in NSW occurred at residential premises (84.9%; 353,651) followed by outdoor/public places (7.8%; 32,447), business/commercial (2.3%; 9,398) and licensed premises (1.3%; 5,589). Further examination of abuse types that occurred within residential premises found that assault (unspecified) (34.4%; 121,781) was the most common abuse type, followed by verbal abuse (24.0%; 84,715), and punching (16.5%; 58,306) (Figure 2).

### Injury Type

**Figure 3:**
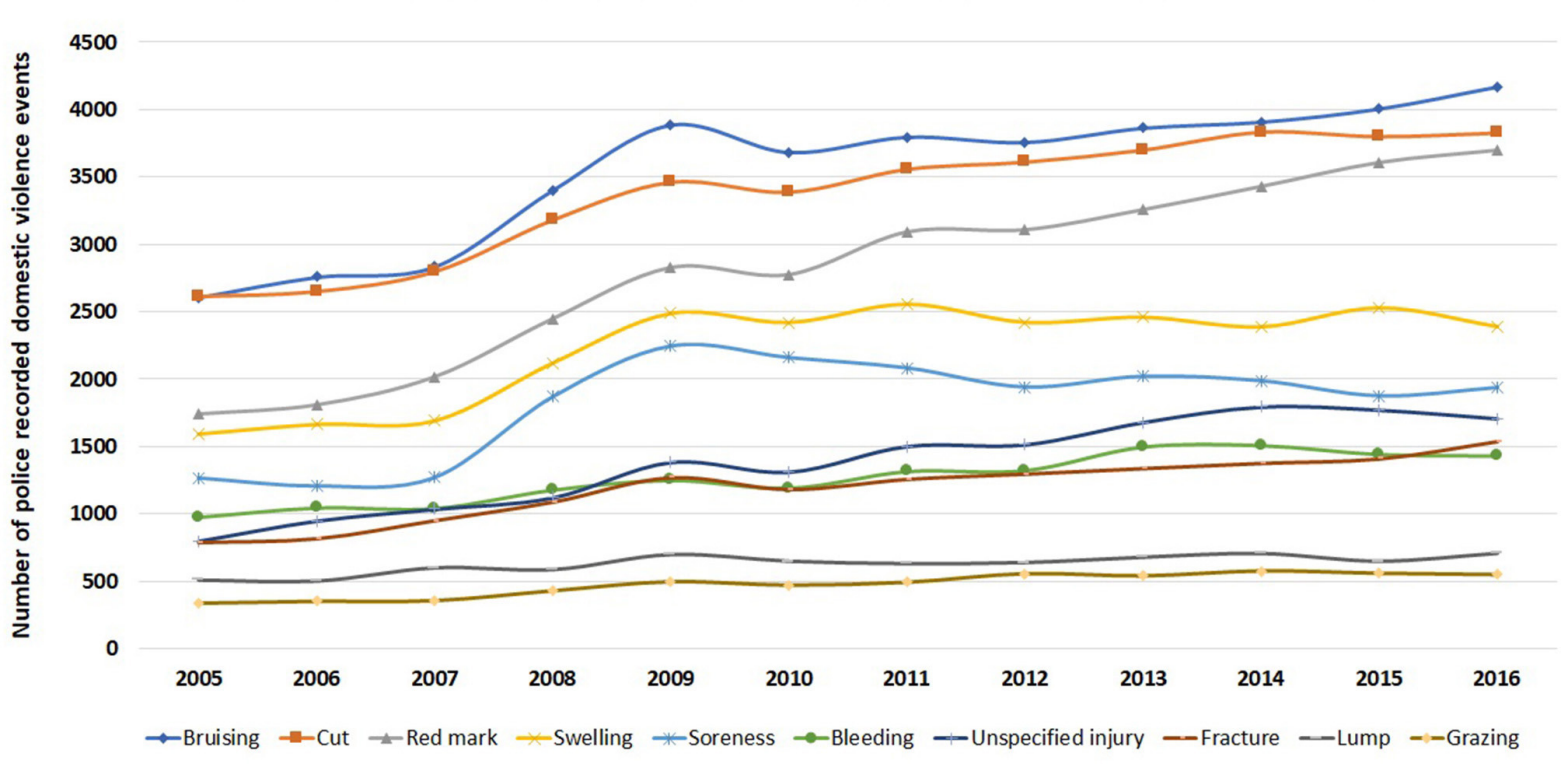
Trend in injury types (top 10 only) for victims in 416,441 police recorded domestic violence events in NSW, January 2005–December 2016.

Over the 12-year period, the most common types of injury were bruising, cuts, red marks (on the skin), swelling, and soreness (Figure 3). There was a significant increase (*P* < 0.001; degree of freedom of 11) in all the common injury types from 28.3% in 2005 to 35.6% of events which recorded an injury type in 2016.

**Figure 4:**
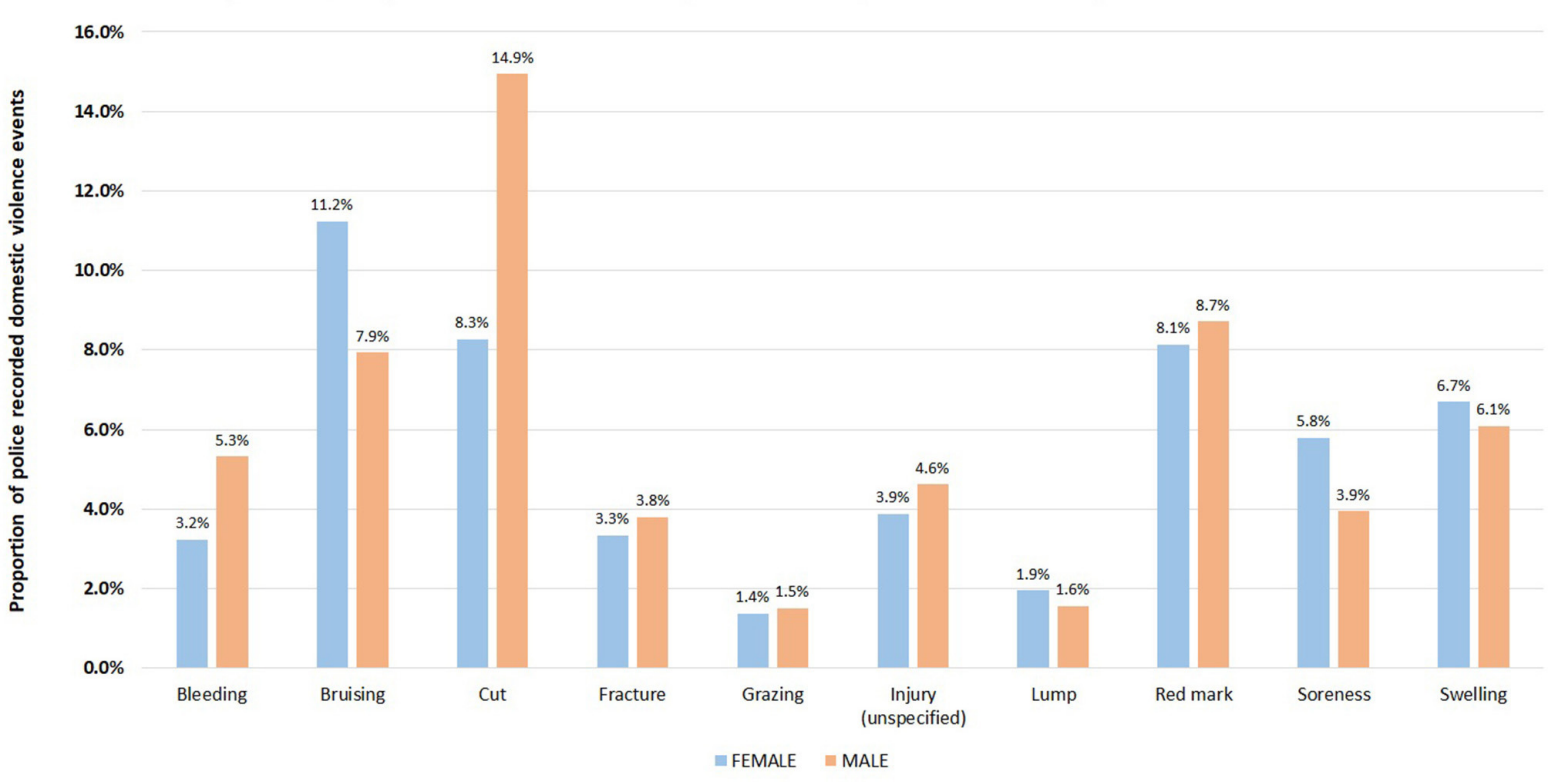
Comparison of proportions of injury types (top 10 only) for male and female victims in 416,441 police recorded domestic violence events in NSW, January 2005–December 2016.

The pattern of injuries sustained by victims in domestic violence events differed between males and females (Figure 4). Cuts and bleeding were more commonly observed in male victims than female victims (14.9% [14,373 events] vs. 8.3% [25,737] for cuts; and 5.3% [5,118] vs. 3.2% [10,041] for bleeding; degree of freedom 1). Bruising was more commonly recorded for female victims that male victims (11.2% [34,945 events] vs. 7.9% [18,008]; degree of freedom of 1).

### Abuse Types

**Figure 5:**
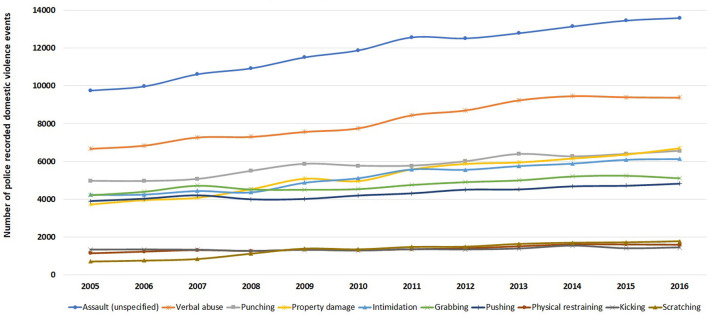
Trend in abuse types (top 10 only) in 416,441 police recorded domestic violence events in NSW, January 2005–December 2016.

Overall, 294,024 of 416,441 police recorded domestic violence events had at least one reported abuse type between 2005 and 2016 ranging from 66.3% (in 2005) to 72.5% (in 2013) (*P* < 0.001; degree of freedom of 11). Assault (unspecified) was the most common abuse type recorded, occurring in more than 10,000 events each year (Figure 5).

**Figure 6:**
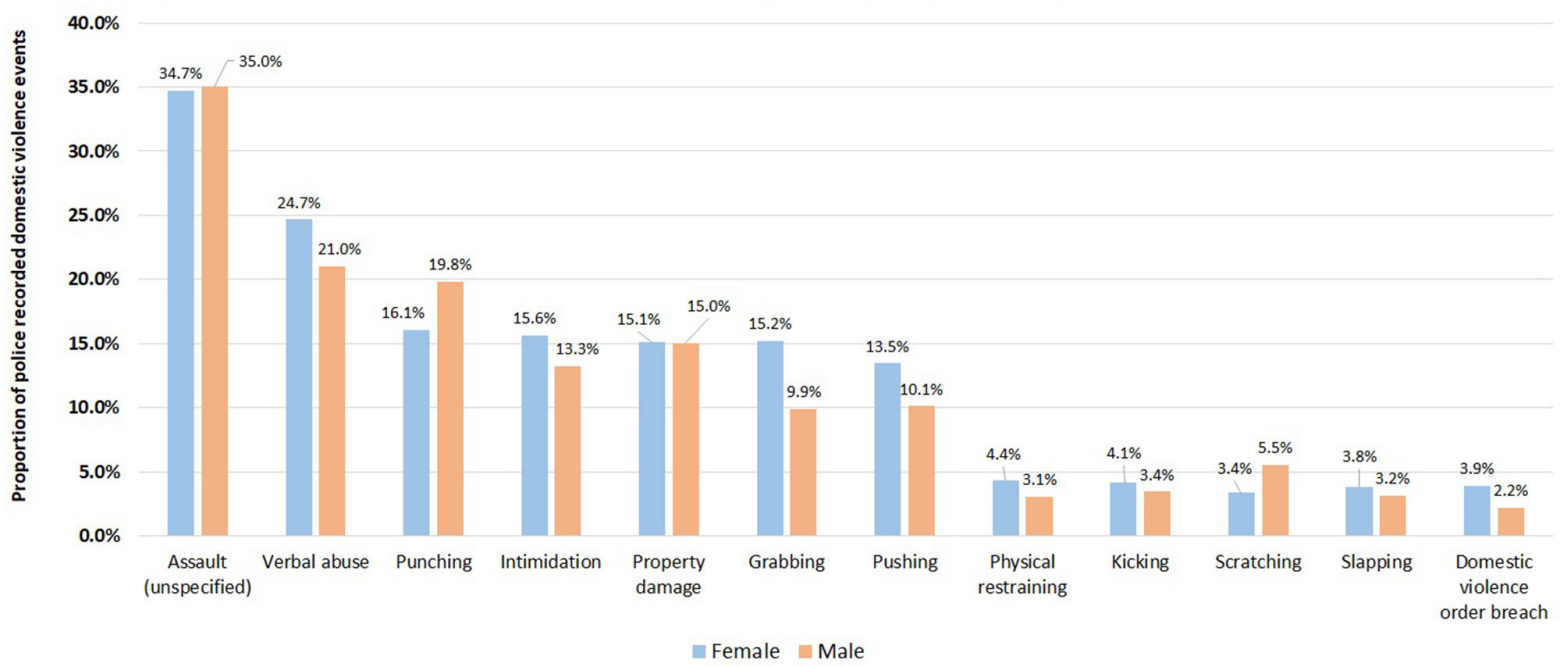
Comparison of proportions of abuse types (top 10 only) for male and female victims in 416,441 police recorded domestic violence events in NSW, January 2005–December 2016.

**Figure 7:**
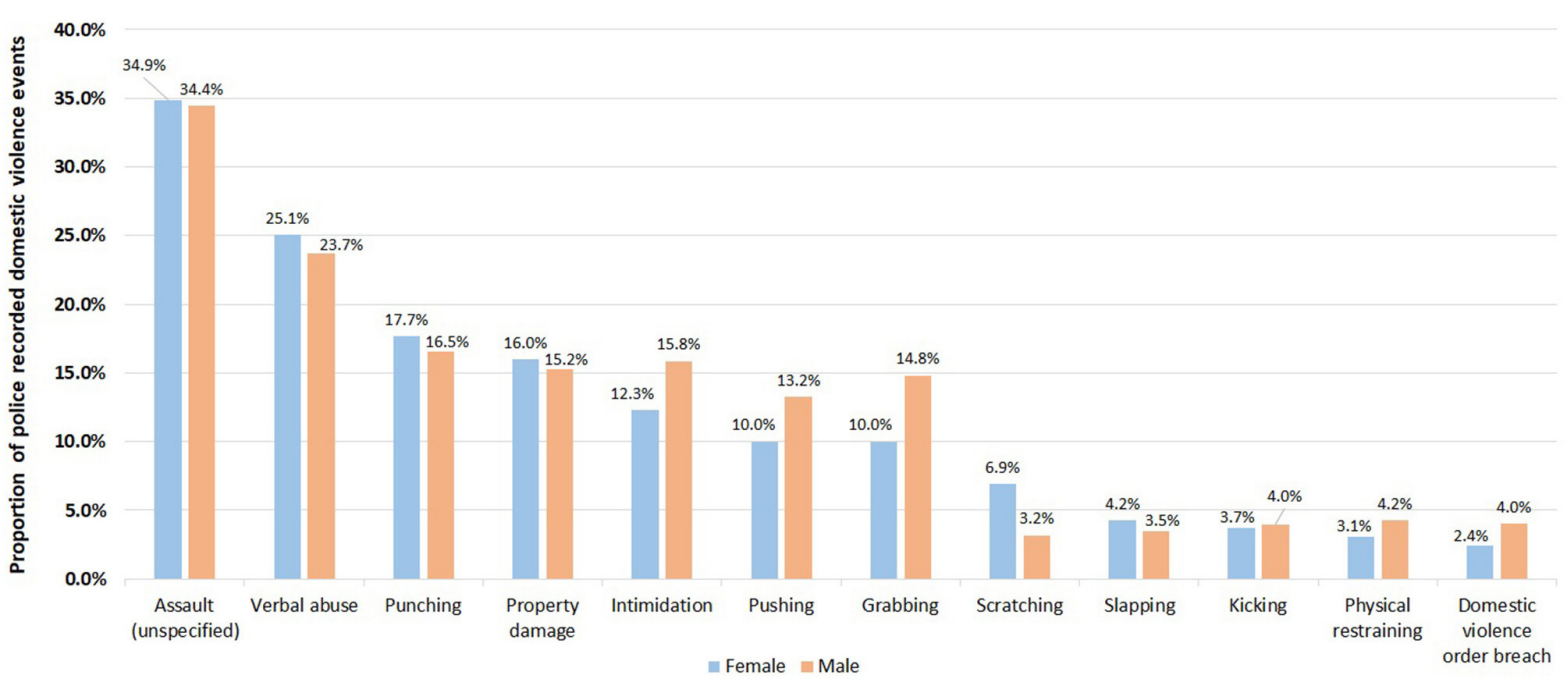
Comparison of proportions of abuse types (top 10 only) for male and female POIs in 416,441 police recorded domestic violence events in NSW, January 2005–December 2016.

Abuse types differed between male and female victims (Figure 6) with women more likely than men to experience hands-on abuse such as grabbing (15.2% vs. 9.9%, *P* < 0.001; degree of freedom of 1) and pushing (13.5% vs. 10.1%, *P* < 0.001; degree of freedom of 1) (Figure 6). Male victims were more likely to be subjected to punching compared to female victims (19.8% vs. 16.1%, *P* < 0.001; degree of freedom of 1) (Figure 6). Female POIs were more likely to scratch the victim than male POIs (6.9% vs. 3.2%, *P* < 0.001; degree of freedom of 1); while male POIs are more likely to conduct intimidation compared to female POIs (15.8% vs. 12.3%, *P* < 0.001; degree of freedom of 1), or grab a victim (14.8% vs. 10.0%, *P* < 0.001; degree of freedom of 1) (Figure 7).

### Relationship Status

Myriad relationship dynamics exist between victims and POIs in the context of domestic violence (Supplementary Figure 1). Of the 170,970 female victims, around three quarters of relationships were spouse/partner (29.1%; 49,758), boyfriend/girlfriend including ex-boyfriend/girlfriend (25.7%; 43,949), and ex-spouse/ex-partner (19.6%; 33,501). Among the 73,088 male victims, the most common relationships with a POI were: parent/guardian of the victim (13.3%; 9,985), boyfriend/girlfriend including ex-boyfriend/girlfriend (13.3%; 9,735), and other family member (13.2%; 9,615) (Supplementary Figure 1).

### Mental Illness

We previously reported on the extraction of mental illness mentions from police narratives using text mining and their subsequent classification into the ICD-10 framework (16, 19, 24) (Supplementary Table 2). Here we report on trends in mental illness mentions over time to highlight how these data can be used for surveillance of mental health in domestic violence as well as the most common mental illnesses for POIs and victims at the ICD-10 level 2.

**Figure 8:**
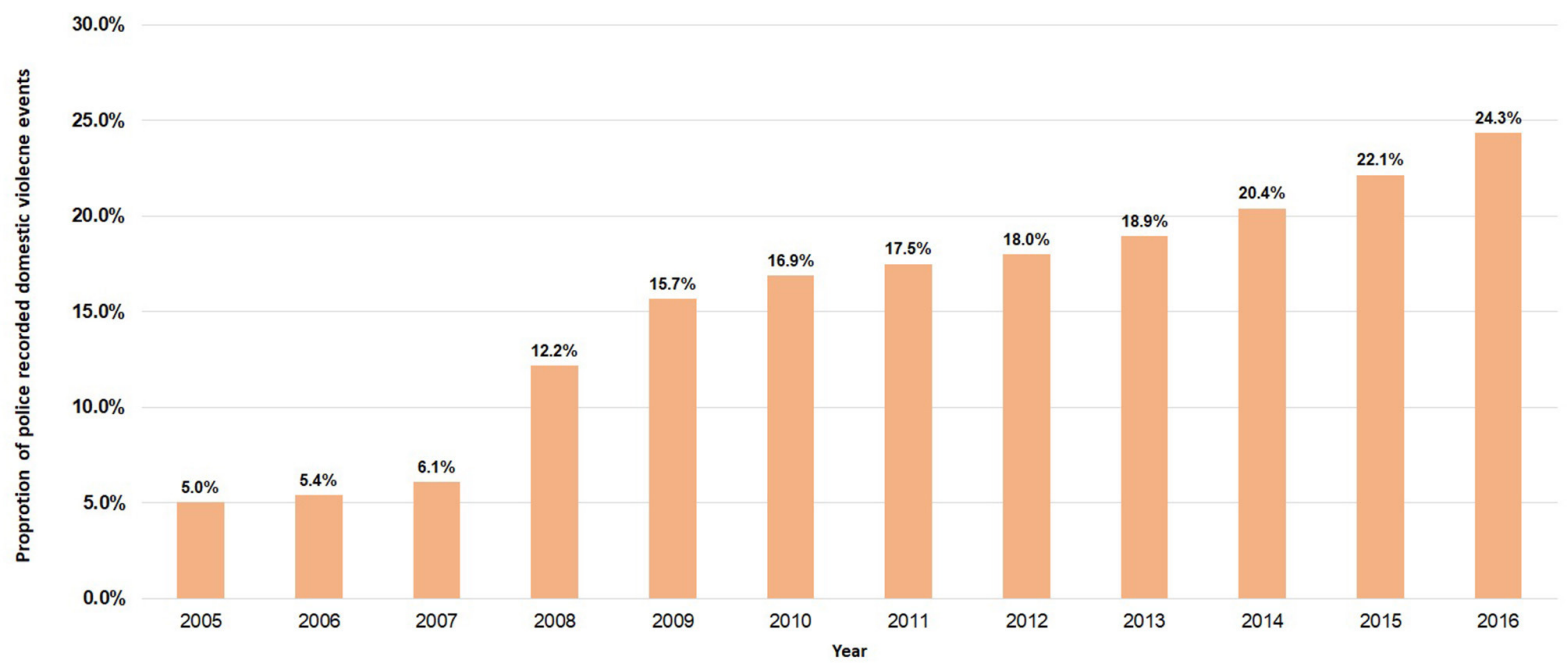
Trend in the proportion of police recorded domestic violence events (*N* = 416,441) that reported a mental illness in NSW, January 2005–December 2016.

**Figure 9:**
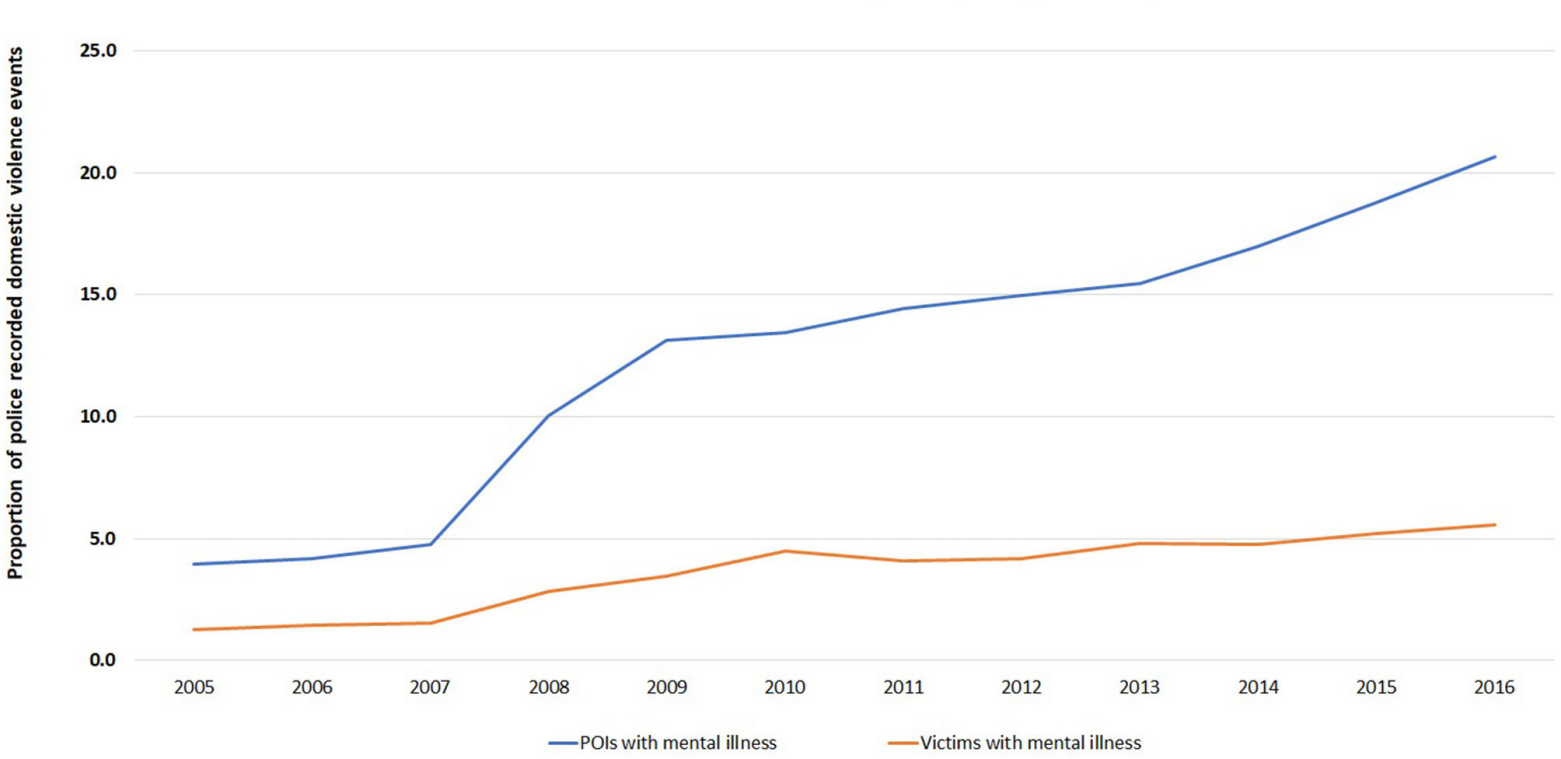
Trend in the proportion of police recorded domestic violence events that had a mental illness for either a POI or a victim in NSW (*N* = 64,587), January 2005–December 2016.

A total of 64,587 events between 2005 and 2016 had a mention of a mental illness for either the POI or victim. Overall, there was an increase in the proportion of events in which mental illness was recorded over time (Figure 8, *P*_trend_ <0.001; degree of freedom of 11). By 2016, one in four events (24.3%) had a mention of a mental illness compared to 5.0% in 2005. The proportion of domestic violence events with a recorded mental illness has also increased most for POIs between 2005 and 2016 compared with victims (Figure 9, *P*_trend_ <0.001 for both; degree of freedom of 1).

#### POI Mental Illness

The most commonly recorded mental illnesses were similar for female and male POIs. However, male POIs were observed to have been associated with behavioral and emotional disorders with onset usually occurring in childhood and adolescence (Table 1, *P*_trend_ <0.001; degree of freedom of 11) while female POIs tended to be related with a steady rise in anxiety disorders (1.1% in 2005 to 17.5% in 2016) (Table 1, *P*_trend_ <0.001; degree of freedom of 11). Depression was the most common mental illness recorded in POIs with increasing rates across the 12-year period (10.8–26.6% for males vs. 22.6–33.9% for females) (Table 1). Interestingly, alcohol abuse in male POIs had the highest proportion of domestic violence events between 2005 and 2008 (25.2–26.4%) but decreased over time (12.4% in 2016) (Table 1). Similarly, alcohol abuse was observed to decline over time for female POIs from 19.2% in 2005 and to 11.6% in 2016 (Table 1).

**Table 1:** Number and trends in the proportion of events with the most common mental illnesses for male and female POIs and victims in 416,441 police recorded domestic violence events that occurred in residential premises in NSW, January 2005–December 2016.

	**Mental illness**	**2005**	**2006**	**2007**	**2008**	**2009**	**2010**	**2011**	**2012**	**2013**	**2014**	**2015**	**2016**
Male POIs	Alcohol abuse	175 (25.2%)	153 (22.1%)	193 (23.8%)	360 (26.4%)	379 (21.8%)	372 (19.7%)	345 (16.4%)	299 (13.5%)	317 (13.6%)	366 (13.6%)	394 (12.2%)	453 (12.4%)
	Bipolar disorder	46 (6.6%)	54 (7.8%)	76 (9.4%)	161 (11.8%)	217 (12.5%)	225 (11.9%)	280 (13.3%)	310 (14.0%)	329 (14.1%)	400 (14.9%)	442 (13.7%)	523 (14.3%)
	Major depressive disorder, single episode	75 (10.8%)	80 (11.6%)	110 (13.5%)	228 (16.7%)	323 (18.5%)	355 (18.8%)	434 (20.6%)	460 (20.7%)	459 (19.6%)	604 (22.4%)	788 (24.4%)	969 (26.6%)
	Other behavioral and emotional disorders with onset usually occurring in childhood and adolescence	104 (15.0%)	108 (15.6%)	126 (15.5%)	197 (14.4%)	232 (13.3%)	260 (13.7%)	291 (13.8%)	317 (14.3%)	359 (15.4%)	365 (13.6%)	450 (13.9%)	474 (13.0%)
	Schizophrenia	69 (9.9%)	82 (11.8%)	87 (10.7%)	150 (11.0%)	213 (12.2%)	235 (12.4%)	314 (14.9%)	333 (15.0%)	360 (15.4%)	378 (14.0%)	437 (13.5%)	490 (13.4%)
Female POIs	Alcohol abuse	34 (19.2%)	51 (23.4%)	54 (22.5%)	65 (17.8%)	76 (15.6%)	94 (17.2%)	77 (12.3%)	83 (12.4%)	95 (13.1%)	92 (11.0%)	116 (13.0%)	121 (11.6%)
	Bipolar disorder	20 (11.3%)	34 (15.6%)	32 (13.3%)	62 (17.0%)	79 (16.2%)	101 (18.5%)	126 (20.1%)	142 (21.1%)	132 (18.2%)	176 (21.1%)	170 (19.0%)	184 (17.7%)
	Major depressive disorder, single episode	40 (22.6%)	55 (25.2%)	58 (24.2%)	104 (28.5%)	147 (30.1%)	164 (30.0%)	188 (29.9%)	229 (34.1%)	238 (32.8%)	265 (31.7%)	296 (33.1%)	353 (33.9%)
	Other anxiety disorders	2 (1.1%)	6 (2.8%)	9 (3.8%)	13 (3.6%)	25 (5.1%)	33 (6.0%)	50 (8.0%)	50 (7.4%)	64 (8.8%)	106 (12.7%)	121 (13.5%)	182 (17.5%)
	Schizophrenia	21 (11.9%)	24 (11.0%)	21 (8.8%)	34 (9.3%)	51 (10.5%)	50 (9.1%)	76 (12.1%)	68 (10.1%)	73 (10.1%)	94 (11.2%)	75 (8.4%)	107 (10.3%)
Male victims	Alcohol abuse	5 (10.0%)	17 (18.3%)	17 (16.8%)	14 (9.4%)	25 (14.0%)	31 (14.1%)	18 (7.4%)	24 (9.9%)	27 (8.9%)	20 (6.5%)	22 (6.3%)	31 (8.1%)
	Attention deficit hyperactivity disorder	18 (36.0%)	17 (18.3%)	25 (24.8%)	23 (15.4%)	29 (16.3%)	37 (16.8%)	27 (11.1%)	31 (12.8%)	43 (14.2%)	35 (11.3%)	42 (12.0%)	56 (14.5%)
	Bipolar disorder	5 (10.0%)	5 (5.4%)	5 (5.0%)	14 (9.4%)	17 (9.6%)	23 (10.5%)	39 (16.0%)	30 (12.4%)	29 (9.6%)	26 (8.4%)	37 (10.5%)	43 (11.2%)
	Major depressive disorder, single episode	14.0%	11.8%	3.0%	12.8%	16.9%	20.5%	14.4%	12.4%	16.5%	24.8%	19.1%	21.6%
	Schizophrenia	3 (6.0%)	5 (5.4%)	8 (7.9%)	8 (5.4%)	12 (6.7%)	19 (8.6%)	22 (9.1%)	24 (9.9%)	41 (13.5%)	28 (9.0%)	39 (11.1%)	36 (9.4%)
Female victims	Alcohol abuse	41 (18.0%)	47 (18.7%)	41 (15.4%)	59 (13.8%)	66 (11.8%)	65 (10.6%)	67 (9.2%)	62 (8.5%)	75 (8.2%)	55 (6.1%)	95 (8.8%)	90 (7.3%)
	Attention deficit hyperactivity disorder	31 (13.6%)	44 (17.5%)	35 (13.1%)	35 (8.2%)	44 (7.9%)	59 (9.7%)	52 (7.2%)	50 (6.9%)	73 (8.0%)	56 (6.2%)	76 (7.1%)	84 (6.8%)
	Bipolar disorder	14 (6.1%)	18 (7.1%)	30 (11.2%)	65 (15.2%)	71 (12.7%)	86 (14.1%)	108 (14.9%)	109 (15.0%)	136 (14.9%)	120 (13.2%)	127 (11.8%)	176 (14.3%)
	Major depressive disorder, single episode	34 (14.9%)	55 (21.8%)	57 (21.3%)	110 (25.7%)	166 (29.8%)	158 (25.9%)	205 (28.2%)	207 (28.5%)	256 (28.1%)	297 (32.7%)	337 (31.3%)	379 (30.7%)
	Other anxiety disorders	14 (6.1%)	17 (6.7%)	27 (10.1%)	34 (7.9%)	60 (10.8%)	63 (10.3%)	82 (11.3%)	118 (16.2%)	130 (14.3%)	173 (19.0%)	243 (22.6%)	306 (24.8%)

#### Victim Mental Illness

Following a similar pattern to POIs, the most commonly reported mental illnesses were the same for male and female victims with the exception that female victims were more likely to have anxiety disorders as opposed to male victims who were more likely to have schizophrenia (Table 1, *P*_trend_ <0.001; degree of freedom of 11). Depression was the most common condition for female victims with a notable increase from 14.9% in 2005 to 30.7% in 2016 (Table 1), with a smaller increase in male victims (14.0% in 2005 to 21.6% in 2016) (Table 1). While illnesses such as attention deficit hyperactivity disorder and alcohol abuse decreased over time in female victims, anxiety disorders increased, particularly from 2011 to 2016. Alcohol abuse was noted to have similar levels in female and male victims throughout the 12-year period (e.g., 7.3% vs. 8.1% in 2016). However, the proportion of events reporting attention deficit hyperactivity disorder was almost double for male victims when compared to female victims across all years (e.g., 14.5% vs. 6.8% in 2016).

## Discussion

Employing text mining on almost half a million event narratives of police recorded domestic violence events over a 12-year period enabled the extraction of significant insights into the epidemiology of domestic violence in NSW. These can be of benefit to the ongoing surveillance and monitoring of domestic violence and provide input into developing prevention and intervention strategies as well as improve the management of domestic violence by first responders such as the police.

Current gaps in data on domestic violence both in Australia and internationally have been acknowledged with calls made to enhance existing data collection systems and identify new sources of information to provide a more comprehensive picture (2–4, 26, 27). It is recognized that much domestic violence goes unreported as occurs using traditional survey methods whereby victims may be unwilling to report incidents of domestic violence for a range of reasons (e.g., the perpetrator being present or in close proximity during the time of the interview, victim residing in temporary accommodation to escape family violence or answering survey questions seen as a low priority) (27).

Gaps in data have been identified regarding groups such as LGBTQ+ individuals, people with disabilities, older people, Indigenous Australians, and people from culturally and linguistically diverse backgrounds as this information is often not recorded by current surveillance systems (5, 6). For example, despite recognition of violence against women with disabilities, its incidence remains under-reported due to barriers and risks that complicate effective reporting (5). Further, data collection systems that focus only on violence perpetrated by intimate partners (particularly males) against women overlook information on other relationship combinations (3, 4) whereas hospital-based injury surveillance only include those at the more severe end of the injury spectrum (e.g., fractures, stabbings) which represent a minority of observed injuries (3).

Other reporting systems rely on information arising from courts such as domestic violence charges and convictions (28). However, these reflect judicial outcomes and do not contain information on characteristics such as victim injuries, threats to harm or kill, the cause of the event, abuse types, or mental illness mentions (28)–such details are collected and recorded in police text narratives by officers attending domestic violence events. Harvesting such information can thus, fill many of these identified domestic violence information gaps. The World Health Organization has suggested that the expansion of the existing knowledge base with new insights in domestic violence prevalence, incidence and patterns could be important tools to engage government and policy makers in addressing these issues with improved programs and strategies (26).

We identified a wide range of injury types associated with domestic violence as well different injury patterns between male and female victims (17). Many of the injuries we extracted are unlikely to warrant hospital attendance and thus would be overlooked by surveillance systems that rely on emergency department presentations or hospital admissions. It was estimated that in 2014–2015, nearly 1 in 5 (18%, or 3,400) of more than 19,000 people admitted to hospital for all assault injuries reported that the perpetrator of the assault was a spouse or a domestic partner (4). In addition, a spouse or domestic partner was reported in more than 4 in 10 (45%) hospitalizations of female assault victims–or more than 2,800 cases–compared with fewer than 1 in 20 (4.4% or 560 cases) male assault hospitalizations (4). This finding perhaps warrants the use of domestic violence police narratives as a tool to shape appropriate early intervention policies (e.g., counseling, support from women's groups) that aim to assist victims burdened by domestic violence emotionally and physically without relying on immediate hospitalizations to set in motion both the legal and social support systems.

One important characteristic recorded by the police is mental illness which we previously reported can be automatically extracted using text mining and classified into a validated framework (i.e., the ICD-10 classification) (24) to describe mental illnesses in victims and perpetrators of domestic violence (16, 19). With 16% (64,587) of domestic violence events having either a victim or perpetrator with a mentioned mental illness as well as an increase in the proportion of events with a reported mental illness from 5.0% in 2005 to 24.3% in 2016, this could reflect a greater awareness and recognition of mental illness by the police and better recording of this characteristic. With an almost 20% increase in mental illness recording in police narratives across the 12-year period, mental illness could be a factor in domestic violence (29, 30). While there have been efforts to identify factors associated with the perpetration of domestic violence, its contribution remains unclear (31, 32). There is evidence that in men, mental health is associated with the perpetration of domestic violence, particularly when substance and alcohol abuse is involved (33–35). Evidence also suggests that people with mental illness are at a greater risk of victimization when compared to those without such symptoms (36–40).

The observation that the police record a wide variety of mental illnesses that extends beyond generic terms (e.g., depression, drug abuse) and ranging in severity in both victims and POIs endorses a further investigation of this association. Input by forensic psychiatrists, psychologists, law enforcement personnel and community groups is needed to explain observations such as the decrease in well-known factors of domestic violence (e.g., alcohol abuse in male POIs), the prevalence of depression in both POIs and victims, and the rise of anxiety disorders in female POIs and victims over the 12-year period.

The automatic inspection of almost half a million of police narratives in domestic violence enabled a more comprehensive picture on abuse types highlighting different behaviors inflicted on victims. We found that three out of four domestic violence events (70.9%; 294,024) had an explicit mention of at least one abuse type between 2005 and 2016, which differed depending on the victim's gender. Female victims were more likely to experience “hands-on” abuse (e.g., grabbing 15.2% vs. 9.9% for male victims; pushing 13.5% vs. 10.1% for male victims), whereas male victims were more likely to be punched (19.8% vs. 16.1% for female victims). This could be used by welfare and law enforcement agencies to improve recognition of abuse types and thus leading to early prevention strategies and appropriate resource allocation. The extracted information from the narratives enabled us to examine in greater detail the nature of the abuse that occurred in residential premises which showed most events (84.9%; 353,651) having assault (unspecified) (34.4%; 121,781) as the most common abuse type, followed by verbal abuse (24.0%; 84,715), and punching (16.5%; 58,306).

Given the timeliness of the event narratives (they are being entered into the COPS system within 24 h of the event), and the employment of text mining, the possibility exists of real time domestic violence surveillance based on these data. This contrasts with certain health-based systems whereby diagnostic codes require time to be coded prior to entry into local hospital-based databases and collated at state or national level for reporting purposes. This can mean that reporting and monitoring runs years behind the actual events and thus limits timely policy responses by government agencies. Further, in addition to the utility of text mining for surveillance purposes, the real time extraction of data from the narratives has practical applications for their incorporation into real time risk assessment tools to identify those at risk of immediate harm. Locally, mental illness mentions from the police text narratives (16, 18, 19) are used by the NSWPF as instantaneous input into their CHIMERA system to improve management of the police's response when attending domestic violence events by informing police officers of any previous information related to the mental health for the POI and victim (41). Based on this information, suggestions are made regarding how to best interact with individuals with particular mental health conditions.

With the police often the first statutory service involved in many of the interactions around domestic violence events, the value of this information should not be underestimated. Since there are potential biases in the way that NSWPF records key details of domestic violence events, there is scope for further refinements in the way the information is being collected (42–44). Linking these data to other administrative collections (e.g., health, welfare, housing, and disability) can answer complex questions about service provision, resourcing, as well as the impacts and outcomes of contact with other health and welfare services (45).

Improved training and awareness from attending police officers can assist in the recording of key details of those involved in a domestic violence event beyond routine demographic and spatio-temporal characteristics and potentially capture information for at-risk sub-populations. Examples of this include reports of mental illness in perpetrators and victims, observed injuries not requiring hospitalization, threats, trends in specific abuse types such as non-fatal strangulation and frequency of abuse within a particular setting such as nursing homes (16–21).

To maximize the utility of police data, consistent definitions and criteria for key characteristics are required to improve measurement and the identification of domestic violence across different data collections. This will enable greater clarity on questions such as how family and domestic violence varies by location and which groups are at greater risk (3).

### Limitations

Our findings are based on domestic violence events that involve a single POI and a single victim only due to a limitation of the text mining approach we adopted as a consequence of limited time and resources required to develop a system to address this. There is a possibility that different (or more nuanced) trends might be observed in cases involving multiple POIs against a single victim and vice versa, something that can be explored in future work in this area. For reporting purposes, we chose to focus only on the top five most common mental illnesses at the second level of the ICD-10 schema. While this decision might exclude certain conditions from other levels (e.g., post-traumatic stress disorder), or less prevalent ones from the same level (e.g., cocaine abuse, attention deficit hyperactivity disorder) in our reporting, detailed presentation could use a more complete breakdown of the identified mental illnesses across all levels.

In addition, while the current methodology did not focus on capturing the location of sustained injuries (e.g., lacerations on hands), such information might be instructive for improving the understanding of the event (e.g., as it might suggest defensive wounding) and the scope of domestic violence. Police data, like other systems used to collect domestic violence information have limitations which preclude their use in isolation from other data sources (27). Most prominent are cases where people experience domestic violence which are not reported to the police and other agencies thus leading to underreporting (46). The application of automated methodologies does not guarantee complete accuracy in the identification of key information since the unstructured text can have multiple synonyms of the same concept, misspellings, abbreviations, typographical errors and bear ambiguous meanings (16, 17). As changes to trends in domestic violence may be influenced by the victims' willingness to report events to the police, trends in recorded crime for domestic violence events need to be considered with caution (27). Finally, police text can include unconscious biases in the reporting of key information leading to incorrect identification of perpetrators and victims (47).

## Conclusion

Despite an international growing body of knowledge that attempts to describe the scope, patterns and risk factors that might be associated with domestic violence, many research gaps remain (26). In Australia, this limitation has been widely acknowledged by statutory agencies (2–4, 6). Calls for enhanced data collection and incorporation of additional data sources that might capture lesser-known facets of domestic violence have been made. We have demonstrated that police event narratives contain valuable information that can be extracted using a validated text mining approach and used for surveillance purposes while providing new insights around the scope of domestic violence in Australia. We detected various increases in domestic violence events that record injury types as well as mental illness within a 12-year period with abuse and injury type patterns differing between male and female victims. Information on mental illness mentions for POIs and victims, conducted abuse types and victim injuries at a population-based level can be made available for reporting purposes to complement other surveillance systems, potentially leading to more effective and timely policy responses by social services, domestic violence organizations, women's groups, child protection agencies and law enforcement.

## Data Availability Statement

The data analyzed in this study is subject to the following licenses/restrictions: The dataset contains potentially identifiable demographic information and hence they are not publicly available. Requests to access these datasets should be directed to g.karystianis@unsw.edu.au.

## Author Contributions

GK: study conception, design and initialization, literature review, data collection, application of text mining, statistical analysis, result interpretation, manuscript preparation, and revision. AA: literature review, result interpretation, manuscript preparation, and revision. PWS, IB, and GN: result interpretation and manuscript revision. HW: statistical analysis and manuscript revision. WL: data analysis and manuscript revision. TB: study conception, initialization, design and supervision, and manuscript revision. All authors contributed to the article and approved the submitted version.

## Funding

This study was supported by a Centre for Research Excellence Grant (APP1057492) and an Australian Institute of Criminology/Criminology Research Grant (34/15-16).

## Conflict of Interest

The authors declare that the research was conducted in the absence of any commercial or financial relationships that could be construed as a potential conflict of interest.

## Publisher's Note

All claims expressed in this article are solely those of the authors and do not necessarily represent those of their affiliated organizations, or those of the publisher, the editors and the reviewers. Any product that may be evaluated in this article, or claim that may be made by its manufacturer, is not guaranteed or endorsed by the publisher.
