# Supplementary material for: Surveillance of Domestic Violence Using Text Mining Outputs From Australian Police Records
Source: Front Psychiatry. 2022 Feb 9;12:787792. doi: 10.3389/fpsyt.2021.787792 (PMC8863744; doi:10.3389/fpsyt.2021.787792)
Supplement: Supplementary file 1 [file Data_Sheet_1.docx]

Supplementary Material

## Supplementary Tables

**Table 1.** Identified abuse types from 492,393 police recorded domestic violence event narratives from January 2005 to December 2016 in New South Wales.

| **Abuse category** | **Abuse type** |
| --- | --- |
| Physical assault | Assault (unspecified), biting, blocking, choking, ordered dog attack, dragging, elbowing, attempting to set fire to premises, gagging, grabbing, hair pulling, headbutting, head locking, kicking, kneeing, physical restraining, pulling, punching, pushing, scratching, shaking, slapping, spitting, stabbing, victim being thrown around, limb twisting, attempt to harm a victim with an object or weapon, and hitting the victim with an object or weapon |
| Threat | Intimidation (via body language) or stating explicit threat(s) to physically harm, sexually assault, and self-harm if the victim does not comply |
| Sexual assault | Sexual assault (e.g., rape) |
| Emotional/verbal abuse | Self-harming when the victim does not comply, yelling profanities, and other emotional/verbal abuse |
| Stalking | Stalking, harassment, and forced entry |
| Financial abuse | Financial control (e.g., no access to credit card) |
| Social abuse | Social restriction and prevent/limit child access |
| Unclassified | Apprehended Domestic Violence Order breach, chasing, lunging, other, and possession of personal effects (e.g., phone and car keys) |
| Property damage | Property damage (ranging from breaking an item to causing damage to a house or vehicle) |

**Table 2:** The ICD-10 Mental and Behavioural Disorders schema used to map the extracted mental illness mentions containing three levels (first, second and third).

| **First level** | **Second level** | **Third level** | **Fourth level** |
| --- | --- | --- | --- |
| Mental disorders due to known physiological conditions | Vascular dementia | - | - |
|  | Unspecified dementia | - | - |
|  | Delirium | - | - |
|  | Unspecified mental disorder due to known physiological condition | - | - |
| Mental and behavioral disorders due to psychoactive substance use | Alcohol related disorders | - | - |
|  | Opioid related disorders | - | - |
|  | Cannabis related disorders | - | - |
|  | Cocaine related disorders | - | - |
|  | Other stimulant related disorders | - | - |
|  | Nicotine dependence | - | - |
|  | Other psychoactive substance related disorders | - | - |
| Schizophrenia, schizotypal, delusional, and other non-mood psychotic disorders | Schizophrenia | Paranoid schizophrenia | - |
|  |  | Disorganized schizophrenia | - |
|  |  | Catatonic schizophrenia | - |
|  |  | Undifferentiated schizophrenia | - |
|  |  | Residual schizophrenia | - |
|  |  | Other schizophrenia | - |
|  |  | Unspecified schizophrenia | - |
|  | Schizotypal disorder | - | - |
|  | Delusional disorders | - | - |
|  | Brief psychotic disorder | - | - |
|  | Shared psychotic disorder | - | - |
|  | Schizoaffective | - | - |
|  | Unspecified psychosis not due to a substance or known physiological condition | - | - |
| Mood [affective] disorders | Manic episode | - | - |
|  | Bipolar disorder | Bipolar disorder, unspecified | - |
|  |  | Other bipolar disorders | Bipolar II disorders |
|  | Major depressive disorder, single episode | Postpartum depression | - |
|  | Major depressive disorder, recurrent | Other recurrent depressive disorders | - |
|  | Persistent mood disorders | Cyclothymic disorder | - |
|  |  | Dysthymic disorder | - |
|  |  | Other persistent mood disorders | Disruptive mood dysregulation disorder |
|  | Unspecified mood disorder | - | - |
| Anxiety, dissociative, stress-related, somatoform and other nonpsychotic mental disorders | Phobic anxiety disorder | Agoraphobia | - |
|  |  | Social phobias | Social phobia, generalised |
|  |  | Specific isolated phobias | Arachnophobia |
|  |  |  | Claustrophobia |
|  |  |  | Acrophobia |
|  |  |  | Androphobia |
|  |  |  | Gynaecophobia |
|  |  | Other phobic anxiety disorders | - |
|  |  | Phobic anxiety disorder, unspecified | - |
|  | Other anxiety disorders | Panic disorder | - |
|  |  | Generalised anxiety disorder | - |
|  |  | Anxiety disorder, unspecified | - |
|  | Obsessive compulsive disorders | Hoarding disorder | - |
|  |  | Excoriation disorder | - |
|  |  | Obsessive compulsive disorder, unspecified | - |
|  | Reaction to severe stress and adjustment disorders | Acute stress reaction | - |
|  |  | Post-traumatic stress disorder | - |
|  |  | Adjustment disorders | - |
|  | Dissociative and conversion disorders | Dissociative amnesia | - |
|  |  | Dissociative fugue | - |
|  |  | Dissociative stupor | - |
|  |  | Other dissociative and conversion disorders | Dissociative identity disorder |
|  |  | Dissociative and conversion disorder, unspecified | - |
|  | Somatoform disorders | Somatization disorder | - |
|  |  | Undifferentiated somatoform disorder | - |
|  |  | Hypochondrial disorders | Body dysmorphic disorder |
|  |  |  | Hypochondriasis |
|  | Other nonpsychotic mental disorders | Depersonalization-derealization syndrome | - |
|  |  | Pseudobulbar affect | - |
|  |  | Nonpsychotic mental disorder, unspecified | - |
| Behavioral syndromes associated with physiological disturbances and physical factors | Eating disorders | Anorexia nervosa | - |
|  |  | Bulimia nervosa | - |
|  |  | Other eating disorders | Binge eating disorder |
|  |  |  | Avoidant food intake disorder |
|  | Sleep disorders not due to a substance or known physiological condition | Insomnia not due to a substance or known physiological condition | Primary insomnia |
|  |  |  | Adjustment insomnia |
|  |  |  | Paradoxical insomnia |
|  |  |  | Psychophysiologic insomnia |
|  | Sexual dysfunction not due to a substance or known physiological condition | Hypoactive sexual desire disorder | - |
|  | Abuse of non-psychoactive substances | Abuse of steroids or hormones | - |
| Disorders of adult personality and behavior | Specific personality disorders | Paranoid personality disorder | - |
|  |  | Schizoid personality disorder | - |
|  |  | Antisocial personality disorder | - |
|  |  | Borderline personality disorder | - |
|  |  | Histrionic personality disorder | - |
|  |  | Obsessive compulsive personality disorder | - |
|  |  | Avoidant personality disorder | - |
|  |  | Dependent personality disorder | - |
|  |  | Other specific personality disorders | Narcissistic personality disorder |
|  |  | Personality disorder, unspecified | - |
|  | Impulse disorders | Pathological gambling | - |
|  |  | Pyromania | - |
|  |  | Kleptomania | - |
|  |  | Trichotillomania | - |
|  |  | Other impulse disorders | Intermittent explosive disorder |
|  |  | Impulse disorder, unspecified | - |
|  | Gender identity disorders | Transsexualism | - |
|  |  | Dual role transsexualism | - |
|  |  | Gender identify disorder | - |
|  | Paraphilias | Fetishism | - |
|  |  | Transvestic fetishism | - |
|  |  | Exhibitionism | - |
|  |  | Voyeurism | - |
|  |  | Paedophilia | - |
|  |  | Sadomasochism | - |
|  |  | Other paraphilias | Frotteurism |
|  | Other personalities of adult and personality behaviour | Factitious disorder | - |
|  | Unspecified disorder of adult personality and behavior | - | - |
| Intellectual disabilities | Mild intellectual disabilities | - | - |
|  | Moderate intellectual disabilities | - | - |
|  | Severe intellectual disabilities | - | - |
|  | Profound intellectual disabilities | - | - |
|  | Unspecified intellectual disabilities | - | - |
| Pervasive and specific developmental disorders | Specific developmental disorders of speech and language | Phonological disorder | - |
|  |  | Expressive language disorder | - |
|  |  | Mixed receptive-expressive language disorder | - |
|  |  | Other developmental disorders of speech and language | Childhood onset fluency disorder |
|  |  |  | Social pragmatic communication disorder |
|  | Specific developmental disorders of scholastic skills | Specific reading disorder | - |
|  |  | Mathematics disorder | - |
|  |  | Other developmental disorders of scholastic skills | Disorder of written expression |
|  | Pervasive developmental disorders | Autism | - |
|  |  | Rett’s syndrome | - |
|  |  | Asperger’s syndrome | - |
|  |  | Pervasive developmental disorder, unspecified | - |
|  | Unspecified disorder of psychological development | - | - |
| Behavioral and emotional disorders with onset usually occurring in childhood and adolescence | Attention-deficit hyperactivity disorders | - | - |
|  | Conduct disorders | Conduct disorder, unspecified | - |
|  |  | Oppositional defiant disorder | - |
|  | Emotional disorders with onset specific to childhood | Separation anxiety disorder of childhood | - |
|  | Disorders of social functioning with onset specific to childhood and adolescence | Selective mutism | - |
|  |  | Reactive attachment disorder of childhood | - |
|  |  | Disinhibited attachment disorder of childhood | - |
|  | Tic disorder | Transient tic disorder | - |
|  |  | Chronic motor or vocal tic disorder | - |
|  |  | Tourette's disorder | - |
|  | Other behavioural and emotional disorders | Unspecified behavioural and emotional disorders | - |
| Unspecified mental disorder |  |  | - |
| Other degenerative diseases of the nervous system | Alzheimer’s disease | Alzheimer’s disease, unspecified | - |
|  | Other degenerative diseases of the nervous system, not elsewhere classified | Frontotemporal dementia | - |
| Systemic atrophies primarily affecting the central nervous system | Huntington’s disease | - | - |
| Injury of unspecified body region | Injury of unspecified body region | Unspecified injury | Suicide attempt |
| Symptoms and signs involving cognition, perception, emotional state and behavior | Symptoms and signs involving emotional state | Other symptoms and signs involving emotional state | Homicidal and suicidal ideations |
| Chromosomal abnormalities, not elsewhere classified | Down syndrome | Down syndrome, unspecified | - |
| Intentional self-harm | - | - | - |
| Unspecified diseases of the nervous system | - | - | - |
| Unspecified drug induced disorders | - | - | - |
| Medications - neuroleptics | - | - | - |
| Medications - antipsychotics | - | - | - |
| Medications – anti anxiety | - | - | - |
| Medications – antidepressants | - | - | - |
| Traumatic brain injury | - | - | - |
| Substance abuse | - | - | - |
| Drug prescription abuse | - | - | - |


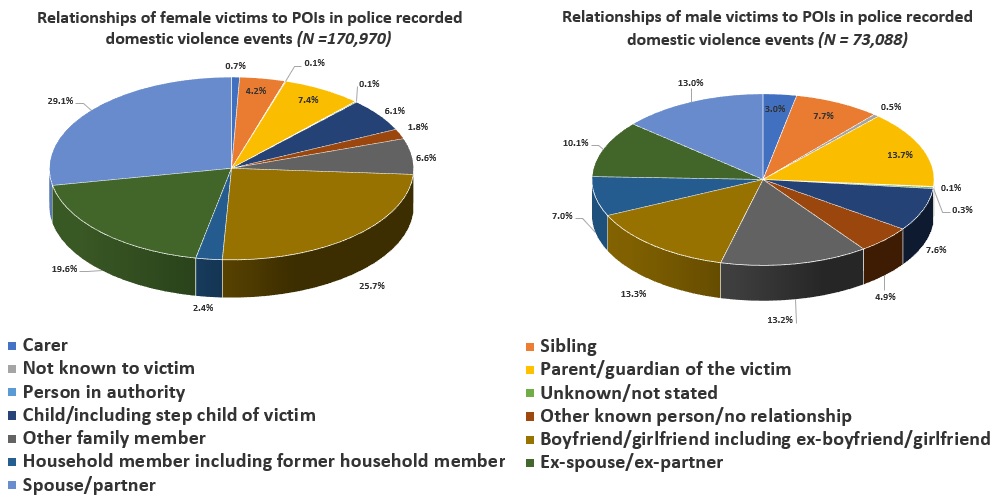


**Figure 1:** Relationship of victims to the POI in 416,441 police recorded domestic violence events by victim gender in NSW, January 2005 - December 2016.
